# Changes in the serum levels of inflammatory cytokines in antidepressant drug-naïve patients with major depression

**DOI:** 10.1371/journal.pone.0197267

**Published:** 2018-06-01

**Authors:** Wei Zou, Renjie Feng, Yuan Yang

**Affiliations:** Department of Neurology and Psychiatry, Tongji Hospital, Tongji Medical College, Huazhong University of Science and Technology, Wuhan, China; Chiba Daigaku, JAPAN

## Abstract

Major depressive disorder (MDD) is a common condition that afflicts the general population across a broad spectrum of ages and social backgrounds. The inflammatory hypothesis of depression posits that immune hyperactivation and dysregulated cytokine production are involved in depression. To investigate cytokine profiles in patients with MDD, we examined the levels of the pro-inflammatory cytokines interleukin (IL)-1β, IL-6, IL-8, and tumor necrosis factor (TNF)-α, and those of the anti-inflammatory cytokines IL-10 and transforming growth factor (TGF)-β1 in antidepressant drug-naïve patients with MDD. Compared to healthy controls, patients with MDD had significantly higher levels of IL-1β, IL-10, and TNF-α, but significantly lower levels of IL-8. There were no significant differences in the levels of IL-6 or TGF-β1. We found linear correlations between IL-1β, TNF-α, and IL-8, and the severity of depression, as well as between IL-8 and anxiety level in patients with comorbid anxiety disorder. In addition, higher IL-1β and TNF-α levels were associated with higher Hamilton Depression Rating Scale (HAMD) scores, while higher IL-8 levels were associated with lower HAMD and Hamilton Anxiety Rating Scale scores. Here we present evidence of changes in cytokine levels in antidepressant drug-naïve patients with MDD. Abnormal expression of inflammatory cytokines in patients with depression suggests that depression activates an inflammatory process. Immunological abnormalities may be involved in the pathophysiology of depression.

## Introduction

Major depressive disorder (MDD) is a common condition that afflicts the general population across a broad spectrum of ages and social backgrounds, and regardless of sex. MDD is a devastating mental illness, and is characterized by a lifetime prevalence of >16% [1]. MDD is also expected to become the second leading contributor to overall disease burden by 2030 [2]. This may lead to overwhelming medical costs. The inflammatory hypothesis of depression posits that immune hyperactivation and dysregulated cytokine production are involved in depression [3]. Cytokines are cell signal transducing proteins or polypeptides that mediate and regulate immune responses and inflammation. They have been shown to cross the blood-brain barrier [4], thereby shaping many aspects of MDD pathophysiology, including neurotransmitter metabolism, neuroendocrine function, and neural plasticity [5]. However, there is clear evidence that changes in cytokine levels are not consistently associated with positive outcomes during depression treatment [6]. Therefore, it has been speculated that inflammation is not generally present in, but only restricted to, particular subgroups of patients with MDD [7, 8].

Antidepressants have a variety of effects on cytokine levels in MDD [6, 9]. However, it is not clear whether these changes in cytokine levels are due to the effects of the antidepressant drugs per se, or whether they are the consequence of psychopathological improvement. Even *in vivo* and *in vitro* studies using the same agent have had different results [10, 11]. Therefore, investigation of drug-naïve patients is essential to elucidating the roles of cytokines in individuals with MDD.

MDD is triggered by stress in a major subgroup of patients. Chronic stress, such as early life stress or separation stress, has been implicated in dysfunction of the hypothalamic-pituitary-adrenal (HPA) axis [12], which blunts the production of serotonin [13]. HPA axis dysregulation appears to be a vulnerability factor for MDD [14, 15]. Pro-inflammatory cytokines, including interleukin (IL)-1β, IL-6, IL-8, and tumor necrosis factor (TNF)-α, influence HPA regulation via mitogen-activated protein kinase signaling [16]. In contrast, anti-inflammatory cytokines, such as IL-1Rα, IL-4, IL-10, and transforming growth factor (TGF)-β1, increase during neuroinflammation induced by psychological or physiological stress. To investigate cytokine profiles in patients with MDD, we examined the levels of the pro-inflammatory cytokines IL-1β, IL-6, IL-8, and TNF-α, and those of the anti-inflammatory cytokines IL-10 and TGF-β1 in antidepressant drug-naïve patients with MDD.

## Materials and methods

### Participants

Patients with MDD were selected from adult outpatients admitted to the Department of Psychiatry at Tongji Hospital (Wuhan, China) between December 2015 and January 2017. Diagnosis of MDD was based on the patients meeting a number of qualitative diagnostic criteria outlined in the *Diagnostic and Statistical Manual of Mental Disorders*, *5th Edition*[17]. Clinical testing included assessments using the Hamilton Depression Scale (HAMD) [18] (HAMD-21; HAMD < 8 [none], HAMD ≥ 8 [mild], HAMD ≥ 17 [moderate], and HAMD ≥ 24 [severe]) and the Hamilton Anxiety Scale (HAMA) [19], which were performed by psychiatrists adhering to good clinical practice in order to minimize variability.

Patients with MDD with other psychiatric comorbidities were excluded. Healthy controls were screened for personal or family histories of neuropsychiatric disorders using the Chinese version of the Mini-International Neuropsychiatric Interview [20] and excluded if such disorders were present. Patients and controls were free of acute and chronic infections, allergies, autoimmune diseases, cancer, and systemic diseases, as determined by self-report, doctor’s report, or physical examination. In addition, patients and controls were excluded if they had a history of immune diseases, chronic infection diseases, tumors, endocrine- or immune-related diseases (e.g., primary adrenocortical insufficiency, renal failure, chronic pancreatitis, primary hypoaldosteronism, carcinomas, hypoparathyroidism, hyperthyroidism, megaloblastic anemia due to iron deficiency, thalassemia, hemochromatosis, liver cirrhosis, Wilson disease, or nephrotic syndrome), antidepressant or antipsychotic treatment, immunomodulatory treatment, analgesic/anti-inflammatory use (e.g., acetylsalicylic acid, ibuprofen, or indomethacin), antibiotic therapy (e.g., hydralazine, tetracyclines, fluoroquinolones, quinolones, calcium, iron, chelating agents, or glucocorticosteroids), or substance abuse. All subjects provided informed written consent. The study protocol was approved by the Ethics Committee of Tongji Hospital, Tongji Medical College, Huazhong University of Science and Technology (institutional review board approval ID: TJ-C20151004).

### Cytokine analysis

Fasting peripheral venous blood samples (5 mL) were collected at 08:00 AM by venipuncture. The samples were left standing for 30 min and then centrifuged (1,006.2 g) for 8 min. A total of 1 mL of the supernatant (serum) was collected for quantitative assays. Serum levels of IL-1β, IL-6, IL-8, IL-10, TNF-α, and TGF-β1 were analyzed using standard capture enzyme-linked immunosorbent assay kits (Xinbosheng Biotechnology). Cytokine analysis was performed using an enzyme marker analyzer (Epoch, BioTeK; Winooski, VT, USA). Detection was performed using a bead-based antibody-antigen sandwich technique. Briefly, specific binding occurred between cytokines and monoclonal mouse anti-cytokine antibodies pre-coated on the enzyme-linked immunosorbent assay plates. Expression levels were determined following incubation with polyclonal anti-cytokine antibodies conjugated to alkaline phosphatase (calf intestine) in 7.5 mL buffer solution. Binding of the secondary antibodies enabled the production of detectable photoelectrons by the luminescent substrate. The results are presented as concentrations of cytokines (pg/mL) in serum. To ensure the accuracy and repeatability of the experiments, all samples were tested in the same run, which included a set of standards measured in duplicate. Our calculations indicated that the coefficients of variability in all groups were less than 10%. The intra-assay and inter-assay coefficients of variability were also lower than 10%.

### Statistical analysis

All statistical analyses in this study were performed using SPSS version 17.0 (IBM Corporation; Armonk, NY, USA) based on a significance level of α = 0.05. For quantitative variables (e.g., IL-1β), mean concentration was used to describe the centralized trend and standard deviation was used to describe the discrete trend. One-way analyses of variance were used to examine quantitative differences between patients and healthy controls. The chi-squared test was used to examine differences in qualitative variables (such as sex, which was described as a percentage) between the two groups. Simple linear correlation analysis was used to estimate correlations between quantitative variables and HAMD or HAMA scores. Multiple linear regression analysis was used to analyze the influences of multiple factors.

## Results

**Fig 1:**
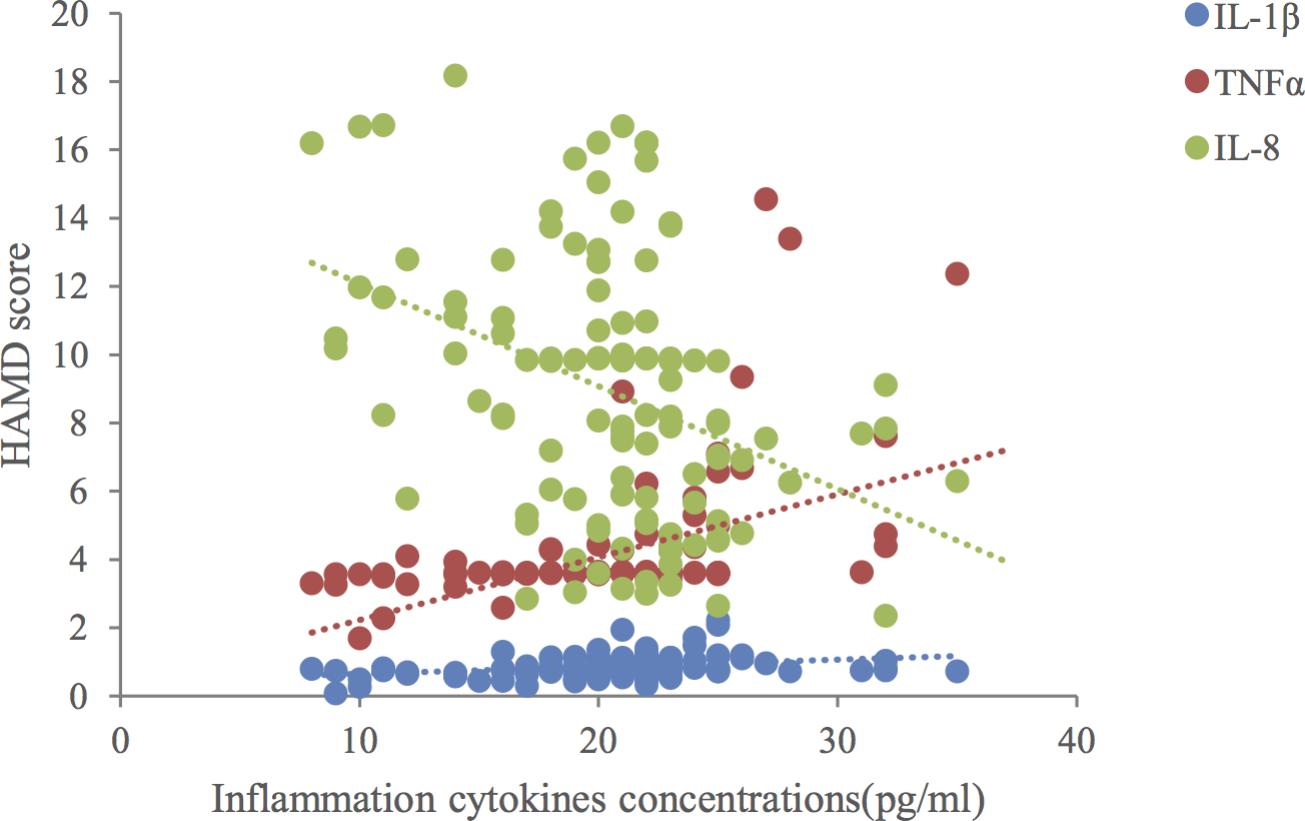
Relationships between interleukin (IL)-1β, IL-8, and tumor necrosis factor (TNF)-α levels, and Hamilton Depression Rating Scale (HAMD) scores.

**Fig 2:**
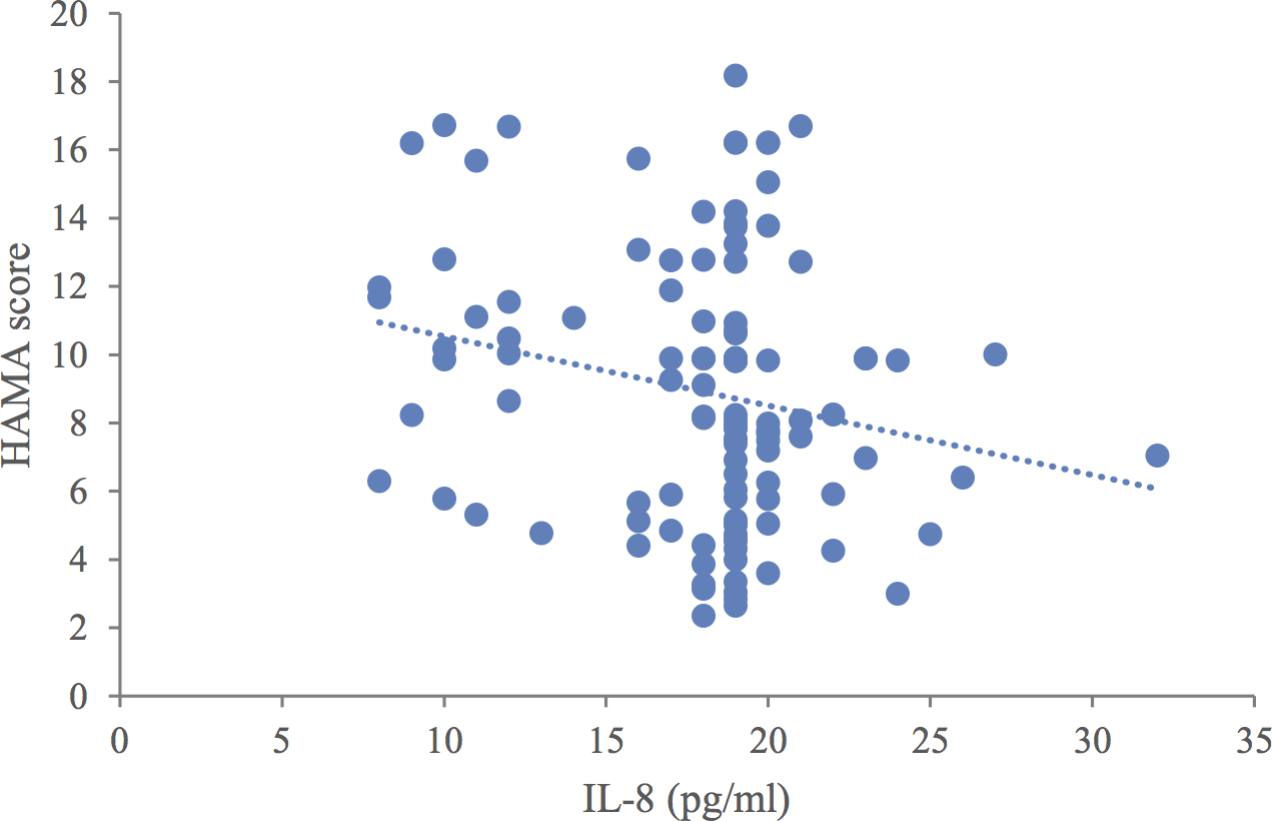
Relationships between interleukin (IL)-8 levels and Hamilton Anxiety Rating Scale (HAMA) scores.

Overall, there were no significant differences in clinical or demographic characteristics between the patients and controls (Table 1). When compared to healthy controls, the patients with MDD had significantly higher levels of IL-1β, IL-10, and TNF-α (*p* < 0.01) and significantly lower levels of IL-8 (*p* < 0.01). There were no significant differences in the levels of IL-6 or TGF-β1 (Table 2). We found linear correlations between IL-1β, TNF-α, and IL-8, and the severity of depression, as well as between IL-8 and anxiety level in patients with comorbid anxiety disorder (*p <* 0.05) (Table 3, Fig 1 and Fig 2). Multiple linear regression analysis revealed that IL-1β, TNF-α, and IL-8 had statistically significant effects on the HAMD score (*p* < 0.05). Higher IL-1β and TNF-α levels were associated with higher HAMD scores, while higher IL-8 levels were associated with lower HAMD scores (Table 4). Multiple linear regression analysis indicated that the IL-8 level had a statistically significant effect on the HAMA score (*p* < 0.05) such that higher IL-8 levels were associated with lower HAMA scores (Table 4).

**Table 1:** Baseline characteristics.

	Major depressive disorder			Healthy controls (n = 102)	*p* value
Characteristic	Mild (n = 20)	Moderate (n = 75)	Severe (n = 22)		
Sex (male/female)	(7/13)	(24/51)	(7/15)	(32/70)	0.99
Age (years)	35.85 ± 12.99	39.2 ± 14.14	35.55 ± 10.48	37.05 ± 11.30	0.49
BMI (kg/m^2^)	21.55 ± 1.93	21.89 ± 2.89	20.60 ± 2.52	23.37 ± 3.12	0.58
Education (years)	11.60 ± 3.12	11.89 ± 3.10	11.77 ± 2.81		0.93
Disease course (months)	26.35 ± 9.06	25.63 ± 16.64	26.91 ± 10.85		0.86
HAMD score (points)	12.70 ± 2.72	20.75 ± 1.72	26.82 ± 3.30		<0.01*
HAMA score (points)	12.75 ± 4.17	19.11 ± 2.70	18.82 ± 4.19		<0.01*

Data are presented as mean ± standard deviation unless otherwise indicated. BMI, body mass index; HAMA, Hamilton Anxiety Rating Scale; HAMD, Hamilton Depression Rating Scale

**Table 2:** Multiplex cytokine comparisons between patients with major depressive disorder (MDD) and healthy controls.

Cytokine	MDD			Healthy controls (n = 102)	*p* value
	Mild (n = 20)	Moderate (n = 75)	Severe (n = 22)		
IL-1β (pg/mL)	0.65 ± 0.25	0.87 ± 0.27	1.07 ± 0.44	0.38 ± 0.21	<0.01*
IL-6 (pg/mL)	2.41 ± 2.58	1.61 ± 1.60	1.69 ± 2.34	1.50 ± 1.29	0.18
IL-10 (pg/mL)	0.51 ± 0.36	0.55 ± 0.38	0.49 ± 0.45	0.26 ± 0.23	<0.01*
IL-8 (pg/mL)	11.56 ± 3.28	8.94 ± 3.99	6.45 ± 2.05	18.67 ± 12.12	<0.01*
TNFα (pg/mL)	3.35 ± 0.56	3.79 ± 0.71	6.19 ± 3.36	2.69 ± 1.46	<0.01*
TGF-β1 (pg/mL)	32.37 ± 14.73	32.02 ± 12.97	29.84 ± 13.65	36.13 ± 14.59	0.11

Data are presented as mean ± SD unless otherwise indicated. IL, interleukin; TGF, transforming growth factor; TNF, tumor necrosis factor

**Table 3:** Relationship between cytokine level in patients with major depressive disorder and depression and anxiety scores.

Cytokine	HAMD *p* value	HAMA *p* value
IL-1β	0.001*	0.236
IL-6	0.195	0.693
IL-10	0.788	0.845
IL-8	<0.001**	0.022*
TNFα	<0.001**	0.475
TGF-β1	0.471	0.903

*,**Statistically significant difference. HAMA, Hamilton Anxiety Rating Scale; HAMD, Hamilton Depression Rating Scale; IL, interleukin; TGF, transforming growth factor; TNF, tumor necrosis factor

**Table 4:** Multiple linear regression analyses of Hamilton Depression Rating Scale (HAMD) and Hamilton Anxiety Rating Scale (HAMA) scores.

Variable	HAMD		HAMA	
	*B*	*P*	*β*	*P*
Interleukin-6	-0.462	0.078	-0.167	0.56
Interleukin-1β	4.805	0.001*	0.971	0.518
Interleukin-10	-1.764	0.255	0.761	0.656
Tumor necrosis factor-α	0.858	<0.001**	-0.386	0.059
Transforming growth factor-β1	0.1	0.051	0.066	0.241
Interleukin-8	-0.499	<0.001**	-0.42	0.003*
Age	0.014	0.573	0.015	0.567
Education	-0.127	0.249	-0.02	0.87
Disease course	0.048	0.473	0.057	0.496

*,**Statistically significant difference

## Discussion

Previous research has suggested that inflammation may be an etiological link between psychological stress and MDD [21, 22]. In the present study, we found that the levels of IL-1β, IL-10, and TNF-α were higher, while the IL-8 level was lower in patients with MDD when compared to healthy controls. In contrast, the levels of IL-6 and TGF-β1 were not different between the two groups (Table 2). The degree of depression was associated with the levels of IL-1β, TNF-α, and IL-8 (Fig 1), while the degree of comorbidity between depression and anxiety was associated with the IL-8 level (Fig 2). These results support the suggestion that an imbalance between pro-inflammatory and anti-inflammatory cytokines triggers the onset of depression [23].

Changes in the pro-inflammatory cytokine profiles were inconsistent in our antidepressant drug-naïve patients with MDD. One plausible interpretation of this finding is that the autonomic nervous system may not be implicated as a significant underlying mechanism in MDD. It has been reported that IL-1β can lead to HPA axis over-activation [24]. TNF-α can increase adrenocorticotropic hormone and cortisol concentrations, which may also lead to HPA axis hyperactivity [25]. HPA hyperactivation in turn may disrupt the normal functions of the glucocorticoid receptor (GR) [26]. GR plays an important role in the response to exogenous glucocorticoids [27], which increase the levels of anti-inflammatory cytokines and decrease the levels of pro-inflammatory cytokines. As the levels of inflammatory cytokines were increased, there may be hypofunction of GRs in MDD. Taken together, the above results also suggest the presence of HPA axis hyperactivity. Overall, hyperactivity of the HPA axis has been the most reproducible pathological hallmark in patients with clinical depression [28–30].

We found that the levels of anti-inflammatory cytokines, such as IL-10, increased in the patients with depression (Table 2). Similar studies have also found increased levels of serum IL-10 in patients with depression, which were decreased or unchanged after antidepressant treatment [6,31,32]. IL-10 levels in patients with early-onset depression (<20 years of age) were reported to have no obvious differences with those in healthy controls, but were reported to be elevated in patients with late-onset depression (≥ 20 years of age) [33]. We found a significant increase in IL-10 levels in our middle-aged patient cohort, which had a mean age of 38.65 ± 13.58 years. Dynamic changes in IL-10 levels may thus be associated with the age of onset of depression.

We lower levels of IL-8 in the patients with MDD, and the IL-8 level was associated with the degrees of anxiety and depression in the patients with depression (Table 4). IL-8 is not only a pro-inflammatory cytokine, but also a chemokine. Chemokines mediate the migration of inflammatory cells and inflammatory factors into inflammatory sites and execute the immune response in the acute inflammatory phase. If depression is triggered by the acute inflammatory phase, long duration of the disorder may lead to the release of chemokines [34]. In this study, the mean disease course was 26.35 months in patients with mild depression, 25.63 months in those with moderate depression, and 26.91 months in those with severe depression (all >6 months). We speculate that the lower levels of IL-8 may reflect the exhaustion of chemokines after the acute inflammatory phase. Moreover, chemokines can modulate the functions of central neurotransmitters[35]. Reduced IL-8 levels may attenuate the 5-hydroxytryptamine and dopamine systems, which, in turn, would lead to depression [36]. The change in the IL-8 level was not only associated with depression, but also correlated with anxiety. Zhen et al. have reported that the IL-8 level is significantly correlated with the anxiety score in anxiety-related diseases such as irritable bowel syndrome, such that higher IL-8 levels are associated with higher anxiety scores [37]. However, in our study, the patients with depression and anxiety had lower levels of serum IL-8, which indicated that IL-8 was related to the morbidity of depression with anxiety. The above-described studies suggest that imbalanced IL-8 levels, whether elevated or reduced, may lead to emotional disorders. Consistent with our findings, previous studies indicate that IL-8 levels are decreased in patients with depression and anxiety who attempt suicide [38]

The changes in IL-6 levels were inconsistent in the patients with MDD [31,39], but were not different between the patients and the healthy controls [33]. We have previously reported that patients with elevated serum IL-6 levels early during a stroke have an elevated risk for post-stroke depression, suggesting that IL-6 is associated with the incidence of depression [30]. Other studies have also found increased levels of serum IL-6 in patients with depression, which were decreased or unchanged after antidepressant treatment [6,31,32,40,41]. Another study also reported no difference in IL-6 levels between patients with depression and controls[42].

Similar to IL-6 levels, the expression levels of TGF-β1 in patients with MDD were inconsistent [43, 44]. We did not find significantly altered TGF-β1 levels in our antidepressant drug-naïve patients. Increased TGF-β1 levels have been reported in patients receiving antidepressant drugs, suggesting that TGF-β1 may be related to antidepressant treatment efficacy [45]. Our results indicate that changes in the levels of cytokines (such as IL-1β, TNF-α, and IL-8) were associated with the degree of depression. However, it has been shown that changes in cytokine levels are not consistently linked to positive outcomes during anti-depression treatment [6]. Therefore, we speculate that over-activation of the immune components associated with MDD may not be a precipitating event of the disorder, but rather a process within MDD.

### Limitations

Our study has several limitations, the first of which is the relatively small sample size and the absence of an analysis of the depressive disorder type. Second, although we studied various cytokines in this investigation, our results do not reflect changes in the entire immune network response, such as anomalies in immune cells (T-helper [TH] 1, TH2, and TH3 cells).Future studies should include analyses of TH1, TH3, and other immune cells, which may provide a comprehensive understanding of the changes in the immune network response. Third, there is evidence suggesting that peripheral cytokine levels are associated with cytokine levels in the brain. Increased cytokine levels in the blood of patients who have not undergone surgery are accompanied by increased cytokine levels in cerebrospinal fluid. The expression of central cytokines may also be induced by peripheral cytokines, suggesting that cytokine levels in the blood reflect changes in cytokine levels in the brain, at least to some extent [46,47]. However, due to the presence of the blood-brain barrier, cytokine level changes in the blood may differ from those in the brain. Further study of cytokine changes in cerebrospinal fluid may clarify the relationship between cytokine changes and depression.

## Conclusions

Our findings demonstrate that antidepressant drug-naïve patients with MDD exhibit abnormal immune regulation and immune system activation. We discussed the cytokine hypothesis, which posits that depression is a process of inflammation enhancement. However, not all serum cytokines had increased levels or (necessarily) participated in immune activation in the patients with depression. Nevertheless, immune activation was related to disease severity. Specific mechanisms may be involved in the complex network structure of cytokines.
